# *Mycobacterium tuberculosis*–Specific Antigen Rv3619c Effectively Alleviates Allergic Asthma in Mice

**DOI:** 10.3389/fphar.2020.532199

**Published:** 2020-09-25

**Authors:** Hussain A. Safar, Ahmed Z. El-Hashim, Hanady Amoudy, Abu Salim Mustafa

**Affiliations:** ^1^Department of Microbiology, Faculty of Medicine, Kuwait University, Kuwait City, Kuwait; ^2^Department of Pharmacology and Therapeutics, Faculty of Pharmacy, Kuwait City, Kuwait

**Keywords:** asthma, hygiene hypothesis, ovalbumin, *Mycobacterium tuberculosis*, Rv3619c, Th1, Th2, inflammation

## Abstract

Despite significant advances, asthma remains a cause of premature death, and current treatments are suboptimal. Antigen-specific Th2 cells and their cytokines are primary mediators of the pathophysiological changes seen in asthma. Studies in animal models have shown that mycobacteria can suppress the asthma phenotype by alteration of the Th1/Th2 cytokines ratio. In this study, utilizing a Th1 delivery system to modulate the allergic airway inflammation in a Th2-driven model of asthma, we evaluated the efficacy of immunization with *Mycobacterium tuberculosis*-specific antigen Rv3619c, either alone or in combination with low dose dexamethasone. The *rv3619c* gene was cloned in an expression plasmid pGES-TH-1, expressed in *Escherichia coli*, and the recombinant protein Rv3619c was purified to homogeneity using affinity chromatography. Mice were immunized with the recombinant protein emulsified in Freund’s Incomplete Adjuvant (IFA) alone and in combination with low dose dexamethasone, and then challenged with ovalbumin (OVA). Airway inflammation was assessed by quantifying airway cytology, histological changes and Th2 cytokine (IL-5) secretion from splenocytes. OVA-specific IgE, IgG and IgG1 from sera was assessed, as well as pERK1/2 expression in the lung tissue. Immunization with recombinant Rv3619c alone inhibited the OVA-induced increase in total cell counts, eosinophil airway cell infiltration in BAL fluid, perivascular and peribronchial inflammation and fibrosis, and goblet cell hyper/metaplasia. In addition, Rv3619c/IFA inhibited the OVA-induced IL-5 in spleen cells, OVA-specific IgE, IgG, and IgG1 levels in sera, and pERK1/2 expression in lung tissue. Immunization with Rv3619c/IFA in combination with low dose dexamethasone resulted in an enhanced effect on some but not all the asthma features. Taken together, this study demonstrates that immunization with Rv3619c/IFA, alone or in combination with dexamethasone, may be an effective treatment strategy for the prevention of asthma.

## Introduction

The incidence of asthma has been increasing globally over the last few decades, with up to 339 million affected people worldwide (particularly children) (Kurvilla et al., 2019). Inhaled corticosteroids (ICS) remain the cornerstone of asthma therapy due to their demonstrated efficacy in reducing airway inflammation (Williams, 2018; Zhang et al., 2018). However, limitations associated with their use still exist (Olin and Wechsler, 2014). For example, long-term usage can result in both local and systemic side effects and patients with severe asthma remain uncontrolled despite receiving high doses of inhaled steroids (Williams, 2018). The introduction of agents such as anti-IgE, anti-IL-4, and anti-IL-5 improved outcomes to a certain degree but more effective and affordable forms of treatment are still needed to deal with a global health challenge (Drick et al., 2018).

Antigen-specific Th2 cells and their cytokines (such as IL-4Ra, IL-5, and IL-13), and inflammatory cells such eosinophils and mast cells are primary mediators of the pathological changes seen in asthma such as airway inflammation and airway hyper-responsiveness (AHR) (Hosoki et al., 2015; Wuhao et al., 2017). Indeed, the Th2 cytokines IL-4 and IL-5 are not only critical for both IgE and eosinophil production, but also for the induction of the overall asthma phenotype and severity (Stokes, 2017; Drick et al., 2018). The recent introduction of new biologics that specifically target these molecules recognizes their importance in driving the asthma disease. Indeed, recent studies have shown that when monoclonal anti-IgE omalizumab, or the anti-IL-5 mepolizumab are added to the asthma therapy, there is a reduction in the frequency of asthma exacerbation and hospitalization and the necessity for higher doses of ICS (Stokes, 2017; Drick et al., 2018). Th17 cell cytokines such as IL-17A and IL-22 have also been reported to enhance neutrophilic airway inflammation and Th2 cell-mediated eosinophilic airway inflammation in asthma, and are thought to contribute to the severity of asthma by enhancing eosinophil recruitment into the airways and mediating Th2-cell differentiation (Wakashin et al., 2008).

In contrast to the pathological roles played by Th2 and Th17 cells and cytokines, cytokines secreted by Th1 and Treg cells may play a protective role in asthma (Ji et al., 2014). This has been elegantly highlighted by several studies demonstrating that Th1 cells can suppress Th2-mediated airway inflammation (Han et al., 2012; Tsujimura et al., 2014). Indeed, inadequate differentiation of naïve T cells into Th1 and Treg cells has been suggested as one of the main reasons for the increase in the prevalence of asthma. This is possibly due to a decrease in infectious diseases because of a general improvement in hygiene practices across the world (Ownby and Johnson, 2009). Several studies have shown that the human immune system tends to deviate to Th2 immune reactions, which are suppressed by Th1 and Treg cells. However, when this suppression is inadequate, Th2 immune reactions are activated which can result in allergic diseases (the so-called “hygiene hypothesis”) (Han et al., 2010). In this context, numerous animal studies have reported that immunization with mycobacterial preparations, including BCG and purified mycobacterial antigens, can prevent or down-regulate asthma features such as AHR and eosinophilic airway inflammation through the induction of Th1/Treg responses. Although live BCG has a suppressive effect on asthma, its use in medical practice may be limited due to adverse reactions it induces (Marciano et al., 2014). BCG has been widely used globally, yet the correlation between BCG vaccination and asthma has had very controversial outcomes possibly due to 1) vaccination with different BCG strains and doses; that may have resulted in variations in the immunogenicity of the vaccine, and 2) in vaccination protocols, particularly the age at which the vaccines are administered; this may influence the function of T-lymphocytes and Th1 activation by BCG and sensitization to allergens (Krishna and Salvi, 2002). To develop a product that suppresses asthma with minimal adverse effects, culture supernatants containing secreted proteins have also been used in the murine model of asthma. Studies have been conducted with native proteins Ag85 Complex, 38 kDa protein, MPB70 purified from the culture supernatants of *Mycobacterium tuberculosis* (Han et al., 2012), and recombinant Ag85B (Tsujimura et al., 2014) to determine the role of individual secreted proteins of *M. tuberculosis* in the immunomodulation of asthma. All of these secreted proteins were shown to be inducers of the protective cytokine interferon- γ (IFN-γ) by Th1 cells (Mustafa and Al-Attiyah, 2009). While the use of purified bacterial proteins should have fewer side effects than whole cell mycobacteria (Han et al., 2012), these proteins are cross-reactive and may face the problem of masking or blocking the anti-asthma effects (Mangtani et al., 2014). The use of *M. tuberculosis*-specific antigens may overcome these limitations. The role of IFN-γ in asthma remains unclear. Some preclinical studies have shown that IFN-γ levels are inversely correlated with Th2 cytokine levels and may indeed have a protective role in asthma (Han et al., 2010; Tsujimura et al., 2014). However, other studies have shown that IFN-γ secretion correlates with asthma severity (Tsujimura et al., 2014; Raundhal et al., 2015). This suggests that the role of IFN-γ in asthma is complex.

In this study we evaluated 1) whether immunization with Rv3619c emulsified with Freund’s Incomplete Adjuvant (IFA), a Th1 delivery system, would inhibit the allergic airway inflammation in a Th2 driven model of asthma, and 2) whether a combination of Rv3619c/IFA with low dose dexamethasone would result in a greater anti-asthma effect than with Rv3619c/IFA immunization alone.

## Materials and Methods

### Plasmid Vectors and Bacterial Strains

The plasmids pGEM-T Easy (Promega corporation Madison, WI, USA) was propagated in *Escherichia coli* strain TOP10 (ATCC, Manassas, VA, USA), and the plasmid pGES-TH-1 (Ahmad et al., 2003) was propagated in *E. coli* strain BL-21 (Novagen, Madison, WI, USA), as described previously (Hanif et al., 2010a). Genomic DNA isolated from *M. tuberculosis* H37Rv (obtained from the American Type Culture Collection, (Rockville, MD, USA) served as the source for the amplification and subsequent cloning of the genes, as described previously (Ahmad et al., 2003). All DNA manipulations, restriction enzyme digestions and bacterial cell transformations with the plasmids were performed according to previously described procedures (Ahmad et al., 2003; Hanif et al., 2010a; Hanif et al., 2010b; Shaban et al., 2013).

### PCR Primers

The primers for amplifications of the target gene from the genomic DNA of *M. tuberculosis* by polymerase chain reaction (PCR) were designed on the basis of nucleotide sequences of these genes in *M. tuberculosis* genome (Tuberculist – Genolist Institute Pasteur, http://genolist.pasteur.fr/TubercuList/). The nucleotide sequences of forward (F) and reverse (R) primers were F: 5’ aatcggatccatgaccatcaactatcaattcggg 3’ and R: 5’ acgaagcttggcccagctggagccgacggcgct 3’ (The restriction sites for *Bam*H I and *Hind* III are underlined in the forward and reverse primers, respectively). The primers contained additional sequences at the 5’ end for digestion with the appropriate restriction enzyme to clone efficiently the PCR-amplified DNA in the expression vector pGES-TH1, as described previously (Ahmad et al., 2003; Hanif et al., 2010a; Hanif et al., 2010b; Shaban et al., 2013). The primers were synthesized commercially (ThrmoFisher Scientific, Ulm, Germany).

### Cloning of Genes in Various Vectors

DNA corresponding to *rv3619c* gene was amplified by PCR using genomic DNA isolated from *M. tuberculosis* and gene-specific primers, as described previously (Ahmad et al., 2003; Hanif et al., 2010a; Hanif et al., 2010b). The amplified DNA was ligated to the cloning vector pGEM-T Easy and propagated in *E. coli* TOP10. The identities of genes cloned in pGEM-T Easy were determined by restriction digestion and DNA sequencing according to standard procedures (Shaban et al., 2013). The DNA fragment corresponding to the amplified gene was restriction digested from the recombinant pGEM-T Easy and subsequently cloned into the expression vector pGES-TH1, as described previously (Ahmad et al., 2003; Hanif et al., 2010a; Hanif et al., 2010b; Shaban et al., 2013).

### Recombinant Protein

The expression vector pGES-TH1 was used for high level expression of Rv3619c fusion protein in *E. coli*, as described previously (Ahmad et al., 2003; Hanif et al., 2010a). The expression of fusion protein was determined by western blotting using anti-GST antibodies (Ahmad et al., 2003). The recombinant protein was purified to homogeneity, with removing endoxins, using affinity glutathione S-transferase (GST) and Nickle affinity (Ni-NTA) chromatography techniques and analyzed by SDS-PAGE using standard procedures (Ahmad et al., 2003).

### Experimental Animals

Six to eight weeks old female pathogen-free BALB/c mice were used in this study. All experiments in mice were performed in accordance with the principles of NC3Rs’ ARRIVE guidelines for reporting humane animal research. The experimental protocols were approved by the “Health Science Center Animal Welfare Committee” and the project funding body (approval number YM06/15), and complied with regulations for the animal care and ethical use of Laboratory Animals in the Health Sciences Center, Kuwait University.

### Immunization of Mice with *M. tuberculosis*-Specific Protein Rv3619c

**Figure 1:**
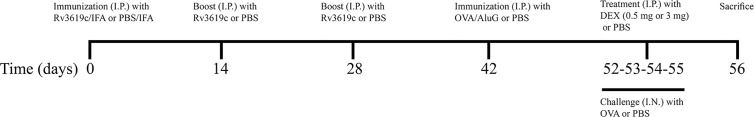
Immunization protocol with Rv3619c/IFA in ovalbumin (OVA)-challenged murine model of asthma. 6–8 weeks ok female balb/c mice (n = 10) were immunized intraperitonealy on day 0 with either 2 µg of Rv3619c emulsified with Freund’s Incomplete Adjuvant (IFA) and boosted with protein only on days 14 and 28 [Rv3619c/IFA and Rv3619c/IFA+ dexamethasone (DEX) (0.5 mg) groups]. All other groups (n = 10) were immunized with PBS emulsified with IFA on day 0 and PBS only on days 14 and 28. All mice (except PBS group mice) were immunized intraperitoneally with OVA/AluG on day 42. Mice were then challenged intranasally with OVA for four consecutive days (days 52 to 55). The PBS group mice were challeneged intranasally with PBS on the same days. Mice in Rv3619c/IFA/dexamethasone (DEX) (0.5 mg), dexamethasone (DEX) (0.5 mg), and dexamethasone (DEX) (3 mg) groups were treated with either dexamethasone an hour prior to intranasal challenge. All mice were sacrificed on day 56 and Bronchoalveolar lavage fluid (BALF) lung tissue, blood and spleens were collected.

Mice in Rv3619c/IFA and Rv3619c/IFA/dexamethasone (0.5 mg) groups (n=10) were immunized intraperitoneally on day 0 with preparations containing 2 μg of purified *M. tuberculosis-specific* recombinant protein Rv3619c emulsified in IFA. The mice were then boosted twice on days 14 and 28 with 2 μg of purified protein only. Mice in PBS, OVA, dexamethasone (0.5 mg) and dexamethasone (3 mg) groups were immunized with PBS emulsified with IFA on day 0 and re-injected with PBS on days 14 and 28 (**Figure 1**).

### Immunization of Mice With Ovalbumin

Mice in all groups (n=10), except for PBS group, were immunized by intraperitoneal injection with 10 μg OVA in 0.2 ml of Aluminum hydroxide Cγ (Alu-Gel-S) [SERVA Electrophoresis GmbH, Heidelberg, Germany] on day 42 (El-Hashim et al., 2012; El-Hashim et al., 2017), and then challenged intranasally, once a day, over 4 consecutive days, with 20 μg OVA in 50 μl PBS on days 52–55. The PBS group mice were injected intraperitoneally with PBS on day 42 and challenged intranasally with 50 μl PBS subsequently over 4 consecutive days (days 52–55) (**Figure 1**).

### OVA Challenge and Dexamethasone Treatment

The mice in the Rv3619c/dexamethasone and dexamethasone (0.5 mg) groups were injected intraperitoneally with 0.5 mg/kg dexamethasone an hour prior to intranasal challenge. Mice in dexamethasone (3 mg) groups were injected intraperitoneally with 3 mg/kg dexamethasone an hour prior to intranasal challenge (**Figure 1**). Mice in other groups were injected intraperitoneally with PBS an hour prior to intranasal challenge. All mice were sacrificed with an overdose of halothane one day after the last intranasal challenge (day 56) (**Figure 1**). Bronchoalveolar lavage fluid (BALF), lung tissue, blood and spleen were collected from each mouse.

### BALF Total and Differential Cell Counts

BALF was collected by cannulating the trachea and washing the lungs with saline solution (4 × 0.3 ml each) (Ji et al., 2014). BALF cells were counted using a particle size counter (Z1 series, Beckman Coulter, Florida, USA) and cytospins (Shandon Scientific Ltd, Cheshire, U.K.) prepared. Cells were stained with Diff-Quik and a differential count of 100 cells was performed using standard morphological criteria (El-Hashim et al., 2012). Results of cell counts in BALF were expressed as total cell count/ml, total differential count/ml, total macrophages/ml, lymphocytes/ml, neutrophils/ml, and eosinophils/ml.

### Histological Changes and Airway Remodeling in the Lung Sections

Lungs were resected and fixed in 10% formalin and embedded in paraffin wax. Sections (3 μm) were stained with H&E and examined for pathological changes under a light microscope. Sections of lungs were also stained with Masson’s Trichrome stain to evaluate fibrillar collagen and connective tissue matrix proteins. Three-micrometer serial sections were mounted on each slide. Mucus and mucus containing goblet cells in the bronchial epithelium were stained with a Periodic Acid–Schiff (PAS) staining. All the slides were blindly scored by at least two different observers to reduce intra-observer variation as described previously (El-Hashim et al., 2017). The average of these scores were calculated for all the animals used in this study. Ten sections from each mouse were assessed. The grade changes description was scored as given below:

- 1 = Normal histology
- 2 = Mild changes
- 3 = Moderate changes
- 4 = Severe changes
- 5 = Highly severe changes

### Th2 (IL-5) Cytokine Levels in Culture Supernatants of Stimulated Spleen Cells

Spleens were collected, and spleen cells were isolated and stimulated with 25 μg OVA, as described previously (Mustafa and Al-Attiyah, 2009; Hanif et al., 2010b). In brief, 5x10^6^ spleen cells were seeded into 96-well tissue culture plates (Nunc, Roskilde, Denmark), stimulated with 25 μg OVA and incubated for 6 days. The negative control wells didn’t receive OVA to test for antigen-nonspecific secretion of cytokines. The culture plates were incubated at 37°C in a humidified atmosphere of 5% CO_2_ and 95% air. The culture supernatants were collected for cytokine assays as described previously (Shaban et al., 2013). The concentrations of Th2 cytokine (IL-5), in the culture supernatants, were estimated using enzyme-linked immunosorbent assay (ELISA) kits purchased from ThermoFisher Scientific Inc, Waltham, MA, USA, as specified by the manufacturer.

### OVA-Specific IgE, IgG, and IgG1 Levels in Sera

Blood was collected from the heart of mice by cardiac puncture and kept at 4°C overnight. Sera was collected after centrifuging blood for 10 min at 4°C. OVA-specific IgE was detected using LEGEND MAX™ MOUSE kit [BioLegend, San Diego, CA, USA], according to the manufacturer’s instructions, and OVA-specific IgG and IgG1 were detected using in-house ELISA (Hanif et al., 2010a). In brief, the wells of each plate were washed four times with 1X washing buffer, and 50 μl of Assay Buffer A was added to standard and sample wells. Fifty microliter of diluted samples (1:10 for OVA-specific IgE and 1:1,000 for OVA-specific IgG and IgG1) were added and the plate was incubated for 2 h at room temperature with shaking on an orbital shaker (Scientific Industries, Inc., New York, USA). The wells were washed, and detection antibody was added, and the plate was then incubated for 1 h at room temperature with shaking. The wells were washed, and Avidin-HRP D or Alkaline phosphatase solution was added. After incubation for 30 min at room temperature, the wells were washed and 100 μl substrate solution F was added. The plate was incubated for 15 min in the dark, the stop solution was added to the wells and the optical density (OD) was read at 450 or 405 nm. The concentration of antibodies was calculated according to mouse OVA-specific IgE, IgG, or IgG1 standards (range from 0.156–10 ng/ml).

### Detecting pERK1/2 Levels in Lung Tissue by Immunofluorescences

Lung sections were incubated in blocking solution (5% BSA + 0.3% Triton X-100 in PBS) for 1 h, followed by incubation overnight at 4° C with primary antibodies (phoso-extracellular signal‐regulated protein kinase ERK1/2 1:50 dilutions) [Cell Signaling Technology, Inc., Danvers, MA, USA] diluted in 1% blocking solution. The sections were washed three times with PBS, and the secondary antibody (Alexa fluor 555 goat anti-rabbit) [SFX kit; ThermoFisher Scientific, Inc., Waltham, MA, USA] was added and incubated at room temperature for 2 h in the dark. The slides were washed three times with PBS, the sections were stained with 4,6 diamidino-2-phenylindole and mounted. Images of the sections were captured on a ZEISS LSM 700 confocal microscope (Carl Zeiss, Jena, Germany) and fluorescence intensities were estimated in defined fields using software Zen 2010D (Zeiss MicroImaging, Carl Zeiss) ZEISS (Khajah et al., 2016; El-Hashim et al., 2017).

### Statistical Analyses

All numerical values were expressed as means ± S.E.M. Total cell counts represent the number of BALF cells/ml. Differential cell counts represent both the absolute number of each cell type/ml of BALF. For histopathology, a semi-quantitative 5-level lung pathology score was used to grade the extent of abnormalities in each microscopic field at 200X. All data were tested for normality. A one-way analysis of variance (ANOVA) test followed by Newman-Keuls *post hoc* test was used to compare mean differences between individual groups for the total and differential cell counts and histopathological data. For the immunofluorescence data, a ANOVA test followed by Bonferroni *post hoc* test was used. The mean difference was considered as significant at a probability level of less than 0.05. All analyses were performed using GraphPad Prism version 7.0 (GraphPad Software, Suite, San Diego, USA).

## Results

### Cloning and Subcloning of Genes and the Expression and Purification of Proteins

The DNA fragment corresponding to *rv3619c* gene was PCR-amplified from the genomic DNA of *M. tuberculosis* using gene-specific primers. The amplified product was purified, ligated to pGEM-T easy vector and propagated into *E. coli* TOP-10 cells and then subcloned into the expression vector pGES-TH1. Western-blot analysis, using anti-GST antibodies, of cell lysates from *E. coli* cells transformed with pGES-TH1/*rv3619c* showed the expression of fusion proteins corresponding to the respective expected size (data not shown). The cells carrying the parent plasmid (pGES-TH1) expressed GST alone and migrated to its expected size (29 kDa) (data not shown).

The induced recombinant *E. coli* cells containing pGES-TH1/*rv3619c* were sonicated and the solubilized proteins were loaded onto Glutathione-Sepharose affinity matrix, cleaved thrombin protease and then reloaded onto Ni-NTA agarose affinity matrix. The eluted protein was analyzed by SDS-PAGE and corresponded to the expected size of GST-free Rv3619c (**Figure S1**).

### Effect of Rv3619c/IFA Alone and in Combination With Dexamethasone on Total and Differential Cell Counts in OVA-Challenged Mice

**Figure 2:**
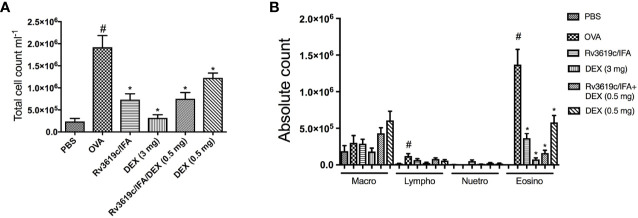
Effect of Rv3619c/Freund’s Incomplete Adjuvant (IFA) alone and in combination with low dose dexamethasone (0.5 mg) treatment on total **(A)** and differential **(B)** cells counts in ovalbumin (OVA)-challenged mice. Immunization of mice with Rv3619c/IFA alone or in combination with dexamethasone (DEX) (0.5 mg) treatment significantly inhibited OVA-induced BAL total cell counts and eosinophilia. Data are expressed as mean ± SEM (n = 10). ^#^P < 0.05 versus time-matched PBS-challenged mice. *P < 0.05 versus time-matched ovalbumin-challenged mice.

Intranasal OVA-challenge resulted in a significant (P<0.05) increase in total BAL cell count and also in eosinophils and lymphocytes, when compared to PBS-challenged control mice (**Figure 2**). Immunization of mice with Rv3619c/IFA resulted in a significant decrease in total cell count and in eosinophil count compared to OVA-challenged/vehicle mice (P<0.05) and was comparable to the high dose dexamethasone treatment (3 mg) (**Figure 2A**). Of interest, immunization of mice with Rv3619c/IFA in combination with dexamethasone (0.5 mg) treatment showed a tendency to decrease the eosinophil infiltration more than that seen with immunization with Rv3619c/IFA alone; however, this was not statistically significant (**Figure 2B**).

### Effect of Rv3619c/IFA Alone and in Combination With Dexamethasone on Histological Changes and Airway Remodeling in the Lung Sections of OVA-Challenged Mice

**Figure 3:**
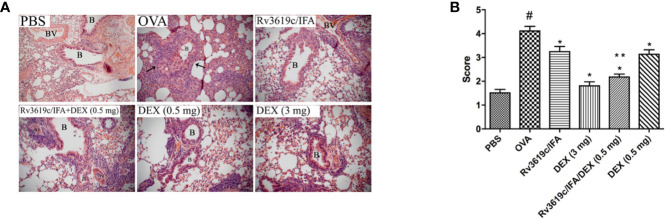
A representative low-magnification light photomicrographs **(A)** and score **(B)** display H&E staining of whole lung samples from PBS-challenged/vehicle, ovalbumin (OVA)-challenged/vehicle, OVA-challenged/Rv3619c/IFA, OVA-challenged/dexamethasone (DEX) (3 mg), OVA-challenged/Rv3619c/IFA/dexamethasone (DEX) (0.5 mg), and OVA-challenged/dexamethasone (DEX) (0.5 mg). OVA-challenged/vehicle treated mice had marked peribronchial and perivascular inflammatory cell infiltrations compared with PBS-challenged/vehicle treated mice. Immunization with Rv3619c/IFA alone resulted in a significant reduction in the peribronchial and perivascular inflammatory cell infiltration compared to OVA-challenged/vehicle treated mice and was comparable with high dose dexamethasone (3 mg). Immunization with Rv3619c/IFA in combination with dexamethasone (0.5 mg) resulted in a greater inhibitory effect on the inflammatory response compared to immunization with Rv3619c/IFA alone. B = bronchioles, BV = blood vessels, (➞) = marked peribronchial and perivascular inflammatory cell infiltrations. Data are expressed as mean ± SEM (n = 10). ^#^P < 0.05 versus time-matched PBS-challenged mice. *P < 0.05 versus time-matched OVA-challenged mice. **P < 0.05 versus Rv3619c/IFA and dexamethasone (0.5 mg).

**Figure 4:**
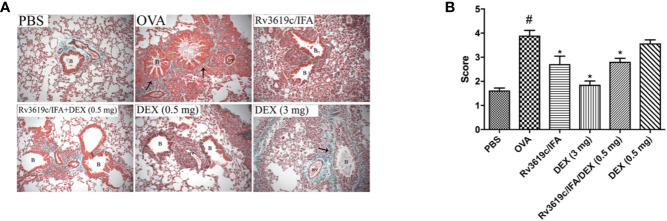
A representative low-magnification light photomicrographs **(A)** and score **(B)** display Masson’s Trichrome staining of whole lung samples from PBS-challenged/vehicle, ovalbumin (OVA)-challenged/vehicle, OVA-challenged/Rv3619c/IFA, OVA-challenged/dexamethasone (DEX) (3 mg), OVA-challenged/Rv3619c/IFA/dexamethasone (DEX) (0.5 mg), and OVA-challenged/dexamethasone (DEX) (0.5 mg). OVA-challenged/vehicle treated mice had marked perivascular and peribronchial fibrosis compared with PBS-challenged mice. Immunization with Rv3619c/IFA alone resulted in a significant reduction in the perivascular and peribronchial fibrosis compared to OVA-challenged/vehicle treated mice and was comparable to the high dose dexamethasone (3 mg). Immunization with Rv3619c/IFA in combination with dexamethasone (0.5 mg) treatment did have any greater effect on the perivascular and peribronchial fibrosis compared to Rv3619c/IFA alone. B = bronchioles, BV = blood vessels, (➞) = marked peribronchial and perivascular fibrosis. Data are expressed as mean ± SEM (n = 10). ^#^P < 0.05 versus time-matched PBS-challenged mice. *P < 0.05 versus time-matched OVA-challenged mice.

**Figure 5:**
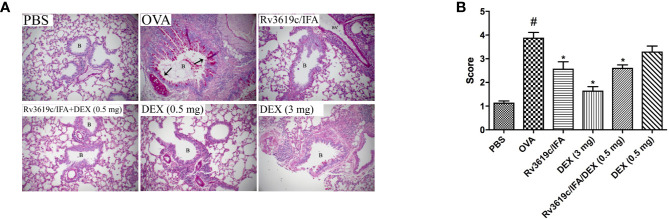
A representative low-magnification light photomicrographs **(A)** and score **(B)** display PAS staining of whole lung samples from PBS-challenged/vehicle, ovalbumin (OVA)-challenged/vehicle, OVA-challenged/Rv3619c/Freund’s Incomplete Adjuvant (IFA), OVA-challenged/dexamethasone (DEX) (3 mg), OVA-challenged/Rv3619c/IFA/dexamethasone (DEX) (0.5 mg), and OVA-challenged/dexamethasone (DEX) (0.5 mg). OVA-challenged/vehicle treated mice had marked bronchial mucus production and goblet cell hyper/metaplasia compared with PBS-challenged mice. Immunization with Rv3619c/IFA alone resulted in a significant reduction in the bronchial mucus production and goblet cell hyper/metaplasia compared to OVA-challenged/vehicle treated mice and was somewhat comparable to the high dose dexamethasone (3 mg). Immunization with Rv3619c/IFA in combination with dexamethasone (0.5 mg) did have any greater effects on the bronchial mucus production and goblet cell hyper/metaplasia compared when compared to Rv3619c/IFA alone. B = bronchioles, BV = blood vessels, (➞) = marked bronchial mucus production and goblet cell hyper/metaplasia. Data are expressed as mean ± SEM (n = 10). ^#^P < 0.05 versus time-matched PBS-challenged mice. *P < 0.05 versus time-matched OVA-challenged mice.

Intranasal OVA-challenge resulted in a marked and significant (P<0.05) perivascular and peribronchial inflammatory cell infiltration and fibrosis (**Figures 3**, **4**), and goblet hyper/metaplasia (**Figure 5**) when compared with PBS-challenged control mice (**Figures 3**–**5**). Immunization of mice with Rv3619c/IFA alone resulted in a significant reduction in all of the histopathological changes compared to OVA-challenged/vehicle mice (P<0.0.5; **Figures 3**–**5**). Interestingly, immunization of mice with Rv3619c/IFA in combination with the low dexamethasone (0.5 mg) treatment resulted in significant enhancement in the reduction of the perivascular and peribronchial inflammation when compared to immunization with Rv3619c/IFA alone and was comparable with the high dose dexamethasone treatment (3 mg) (**Figure 3**). However, no significant decrease in the perivascular and peribronchial fibrosis and goblet cell hyper/metaplasia was observed following immunization of mice with Rv3619c/IFA in combination with the low dexamethasone (0.5 mg) treatment (**Figures 4**, **5**).

### Effect of Rv3619c/IFA Alone and in Combination With Dexamethasone on Th2 Cytokine Secretion in the Spleen of OVA-Challenged Mice

**Figure 6:**
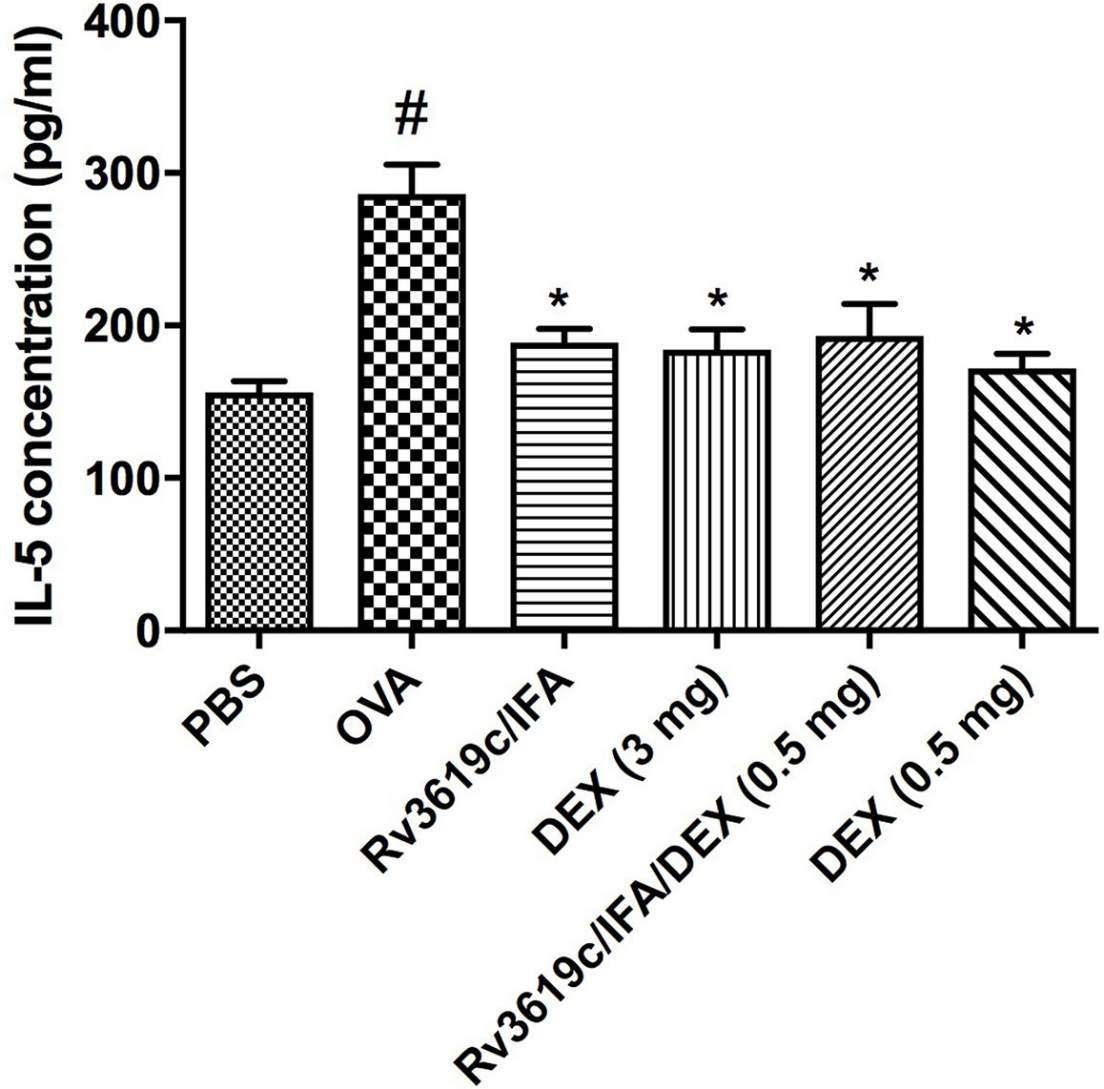
Effect of Rv3619c/Freund’s Incomplete Adjuvant (IFA) alone and in combination with low dose dexamethasone (DEX) (0.5 mg) treatment on Th2 cytokine (IL-5) secretion from spleen cells of ovalbumin (OVA)-challenged mice. Immunization of mice with Rv3619c/IFA showed a significant inhibition of secretion of Th2 cytokine (IL-5). Immunization with Rv3619c/IFA in combination with dexamethasone (0.5 mg) treatment did have any greater effects on the IL-5 levels. Data are expressed as mean ± SEM (n = 10). ^#^P < 0.05 versus time-matched PBS-challenged mice. *P < 0.05 versus time-matched OVA-challenged mice.

Intranasal OVA-challenge induced a significant (P<0.05) increase in the levels of IL-5 secreted from spleen cells when compared to PBS-challenged control mice (**Figure 6**). Immunization of mice with Rv3619c/IFA alone resulted in a significant decrease in IL-5 secretion compared to OVA-challenged/vehicle treated mice (P<0.05) and was comparable to the high dose dexamethasone (3 mg) (**Figure 6**). Immunization of mice with Rv3619c/IFA in combination with dexamethasone (0.5 mg) significantly decreased IL-5 secretions compared to OVA-challenged/vehicle-treated mice but did not result in any further enhancement of the effects of Rv3619c/IFA alone (**Figure 6**).

### Effect of Rv3619c/IFA Alone and in Combination With Dexamethasone on OVA-Specific IgE, IgG, and IgG1 Levels in Sera of OVA-Challenged Mice

**Figure 7:**
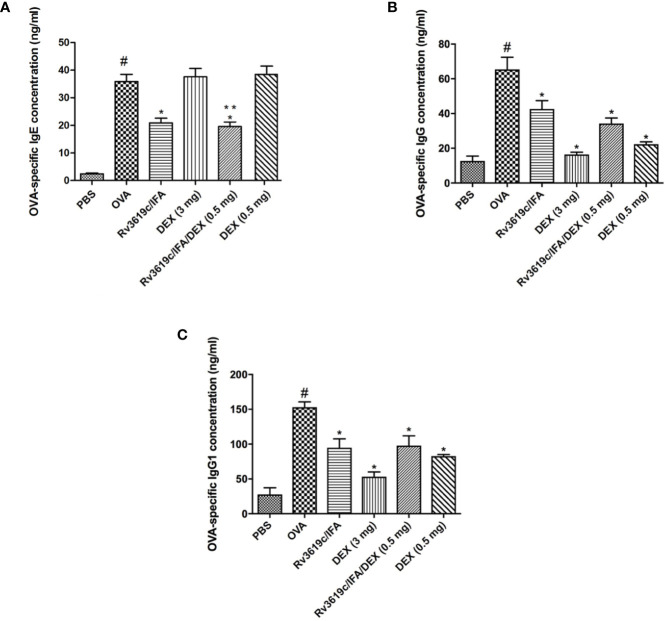
Effect of Rv3619c/Freund’s Incomplete Adjuvant (IFA) alone and in combination with low dose dexamethasone (DEX) (0.5 mg) treatment on sera levels of ovalbumin (OVA)-specific IgE **(A)** IgG **(B)** and IgG1 **(C)** from OVA-challenged mice. Immunization with Rv3619c/IFA alone resulted in significant reduction in the sera levels of OVA-specific IgE, IgG, and IgG1. Immunization with Rv3619c/IFA alone or in combination with dexamethasone (0.5 mg) treatment did have any greater effects on the OVA-specific IgE, IgG, and IgG1. Data are expressed as mean ± SEM (n = 10). ^#^P < 0.05 versus time-matched PBS-challenged mice. *P < 0.05 versus time-matched OVA-challenged mice. **P < 0.05 versus Rv3619c/IFA and dexamethasone (0.5 mg).

Intranasal OVA-challenge induced a significant increase in the sera levels of OVA-specific IgE, IgG, and IgG1 compared with PBS-challenged control mice (**Figure 7**). Immunization with Rv3619c/IFA alone significantly (P<0.05) reduced the sera levels of OVA-specific IgE, IgG and IgG1 compared to OVA-challenged/vehicle treated mice (**Figure 7**). Interestingly, neither low nor high dose dexamethasone treatment had any effect on the OVA-specific IgE levels, however, both doses significantly reduced OVA-specific IgG and IgG1 levels (**Figure 7**).

### Effect of Rv3619c/IFA Alone and in Combination With Dexamethasone on pERK1/2 in the Lung Sections of OVA-Challenged Mice

**Figure 8:**
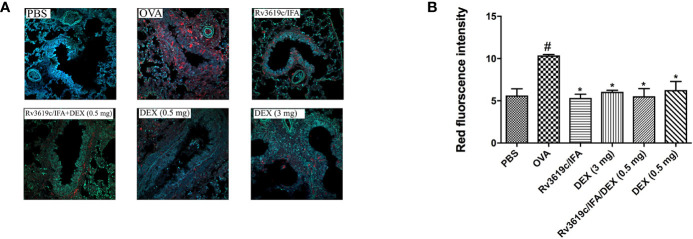
A representative low-magnification light photomicrographs **(A)** and score **(B)** display pERK1/2 fluorescence of whole lung samples from PBS-challenged/vehicle, ovalbumin (OVA)-challenged/vehicle, OVA-challenged/Rv3619c/Freund’s Incomplete Adjuvant (IFA), OVA-challenged/dexamethasone (DEX) (3 mg), OVA-challenged/Rv3619c/IFA/dexamethasone (DEX) (0.5 mg), and OVA-challenged/dexamethasone (DEX) (0.5 mg). OVA-challenged/vehicle treated mice had marked increase in pERK1/2 signals compared with PBS-challenged mice. Immunization with Rv3619c/IFA alone resulted in a significant reduction in the pERK1/2 signals compared to OVA-challenged/vehicle treated mice and was comparable to the high dose dexamethasone (3 mg). Immunization of mice with Rv3619c/IFA in combination with dexamethasone (0.5 mg) did not result in any significant enhancement of the effects of Rv3619c/IFA alone. Data are expressed as mean ± SEM (n = 5). ^#^P < 0.05 versus time-matched PBS-challenged mice. *P < 0.05 versus time-matched OVA-challenged mice.

Intranasal OVA-challenge induced a marked and significant (P<0.05) increase in the levels of pERK1/2 when compared with PBS-challenged control mice (**Figure 8**). Immunization of mice with Rv3619c/IFA alone resulted in a complete and significant (P<0.05) inhibition of the OVA induced increase in the expression of pERK1/2 and was comparable to high dose dexamethasone (3 mg) (**Figure 8**). Immunization of mice with Rv3619c/IFA in combination with dexamethasone (0.5 mg) did not result in any significant enhancement of the effects of Rv3619c/IFA alone (**Figure 8**).

## Discussion

Mycobacterial secretory proteins are known to be strong Th1 inducers and may play an important role in the prevention of asthma in murine models. However, antigens previously reported to show a protective effect were not *M. tuberculosis*-specific, which may decrease their effectiveness in providing effective protection (Mangtani et al., 2014). Our data showed that immunizing mice with a *M. tuberculosis*-specific antigen Rv3619c emulsified with IFA resulted in a significant inhibition of the OVA-induced increase in the total cell count in the BAL, a decrease in eosinophilic and lymphocytic influx into the airways and suppression of the OVA-induced perivascular and peribronchial inflammation, fibrosis and goblet cell hyper/metaplasia. In addition, we showed that immunization with Rv3619c/IFA in combination with treatment with low dose dexamethasone (0.5 mg) had an enhanced inhibitory effect on some of the asthma parameters. These findings suggest that immunization with *M. tuberculosis*-specific antigen Rv3619c may offer a novel approach for the prevention of asthma.

The effects of immunizing mice with Rv3619c/IFA are consistent with data from another murine model of asthma which show that immunization with viral vectors expressing mycobacterial protein Ag85A and Mtb32 reduce allergic inflammatory changes (Zhang et al., 2018). Indeed, immunization with single mycobacterial secretory proteins has been shown to demonstrate a greater inhibitory effect on eosinophil infiltration than live whole-cell BCG (Han et al., 2012). Moreover, our findings are in agreement with other studies demonstrating that immunization of mice with an immunodominant mycobacterial antigen results in anti-inflammatory action and reduction of airway remodeling, resulting in a reduced loss of lung function (Wakashin et al., 2008; Wu et al., 2009; Karamatsu et al., 2012).

Of interest is the finding that immunization of mice with Rv3619c/IFA in combination with a low dose of dexamethasone (0.5 mg) resulted in a greater inhibition of the total cell count and the eosinophil influx in BAL fluid than that noted with immunization with Rv3619c/IFA alone. Moreover, this combination had a significantly greater inhibitory effect on the perivascular and peribronchial inflammation compared to immunization with Rv3619c/IFA alone, and was indeed comparable with the high dose dexamethasone (3 mg). However, there was no enhancement of the inhibition of the goblet cell hyper/metaplasia, fibrosis, IL-5 and IgE levels or ERK1/2 expression over the effects of Rv3619c/IFA alone. These findings suggest that the use of the antigen with low dose steroid combination may be more effective in reducing the asthma phenotype. However, this may not extend to all features of asthma.

Multiple mechanisms are involved in mediating airway inflammation in allergic asthma. The mechanism by which mycobacterial proteins suppress this inflammation in murine models of asthma still remains to be completely established. Based on the so-called “hygiene hypothesis”, the induction of a high Th1 response by mycobacteria or mycobacterial antigens deviates the immune response from the excessive Th2 response, thus inhibiting the development of an allergic asthma-like phenotype (Cookson and Moffatt, 1997). Preclinical studies have demonstrated that Th1 cells contribute to the protection against the development of the allergic asthmatic phenotype (Obihara et al., 2007; Zhang et al., 2012). Indeed, the production of high levels of the Th1 cytokine IFN-γ ameliorates aberrant Th2 responses deviating the immune response toward protective Th1 responses (Han et al., 2010; Zhang et al., 2018). Studies have also shown that Rv3619c protein can induce cellular immune responses by elevating the secretion of Th1 cytokines IFN-γ and IL-12 but not Th2 cytokines such as IL-4 and IL-5 (Mahmood et al., 2011; Safar et al., 2020). In eosinophilic asthma, IL-5 is mainly produced by Th2 cells, mast cells and natural killer T (NKT) cells in the airways. In addition, IL-5 plays a role in promoting eosinophil maturation by inducing the secretions of eosinophil eotaxins 1, 2, and 3 (McBrien and Menzies-Gow, 2017; Pelaia et al., 2017). Furthermore, IL-5 plays an important role in eosinophil migration from the bloodstream to the airways (Shrimanker and Pavord, 2017). In this regard, our data showed that immunization with Rv3619c/IFA alone significantly reduced the OVA elevated levels of IL-5 secreted by spleen cells *in vitro*. Therefore, it is very likely that the anti-asthma effects seen following immunization with Rv3619c may be, in part, due to the increased secretion of IFN-γ. This is consistent with the study of Han and colleagues who showed that immunization with mycobacterial antigens (Ag85 complex, MPB70 and 38-kDa) resulted in a significant increase in the IFN-γ:IL-5 ratio in the spleen compared to the asthma control group (Han et al., 2012). This reduction in IL-5 secretion may be one of the mechanisms by which immunization with Rv3619c, resulted in a reduction in the eosinophilia detected in the BAL fluid and the perivascular and peribronchial eosinophil cellular infiltration. Of interest, despite the protective role that IFN-γ has been shown to have in the prevention of asthma, paradoxically recent studies have demonstrated that IFN-γ can have a pro-inflammatory role in severe asthma (Krug et al., 1995; Raundhal et al., 2015). In murine models of asthma, local expression of IFN-γ in the lung correlated with an increase in allergic airway inflammation, which lead to steroid-resistant airway inflammation by macrophage stimulation (Koch et al., 2006; Kumar et al., 2012). Therefore, it would seem that IFN-γ may play a protective role in early asthma, but once asthma is established, it can contribute to the severity of this disease.

IgE plays an important role in allergic responses by activating receptors in many cell types such as mast cells, eosinophils, dendritic cells and endothelial cells (Matucci et al., 2018). resulting in secretion of numerous inflammatory mediators that cause many pathophysiological changes associated with asthma (Chen et al., 2018). IgE inhibition is a relatively new strategy used in the treatment of asthma, particularly that associated with high serum IgE levels and not very responsive to ICS treatment (Pennington et al., 2016). Clinical studies reported that long-term treatment with anti-IgE therapies, as an add-on to high dose ICS, improves asthma control in both children and adults, reduces the frequency and severity of exacerbations and allows the reduction of doses of ICS (Baena-Cagnani et al., 2015; Mansur et al., 2017). Our findings show that immunization with Rv3619c/IFA significantly inhibited the OVA-induced increase of serum levels of OVA-specific IgE, IgG and IgG1. This could be due to Rv3619c/IFA-induced inhibition of the secretion of Th2 cytokines (Mahmood et al., 2011). In contrast, neither dose of dexamethasone had a positive effect on the elevated serum levels of OVA-specific IgE. The latter results are in line with Zhao et al. who reported that a dose of 1 mg/kg of dexamethasone did not result in the inhibition of OVA-specific IgE levels (Zhao et al., 2006). However, several studies using doses varying from 0.01 to 3 mg/kg dexamethasone have reported a significant reduction in OVA-specific IgE levels (Ma et al., 2014; Zhang et al., 2015; Sharma et al., 2017; Harati et al., 2018). These differences may be related to the difference in the experimental models used.

pERK1/2–dependent signaling is involved in several inflammatory cells such as in mast cell eosinophil differentiation/activation and also in B cell proliferation and survival (Richards et al., 2001; Goplen et al., 2008). pERK1/2 has also been shown to be an important downstream signaling molecule in several of the Th2 cytokine signaling pathways (Alam and Gorska, 2011; Gharagozloo et al., 2013). Indeed increased pERK1/2 expression in airway epithelium and smooth muscle cells has been associated with asthma severity and inhibition pERK1/2 has been shown to be associated significant reduction in the asthma phenotype (Guan et al., 2007; Alam and Gorska, 2011). In this study, our data showed that OVA challenge resulted in a significant increase in pERK1/2 in the lung compared to PBS-challenged control mice, consistent with several other studies (El-Hashim et al., 2012; Kurakula et al., 2015). Immunization of Rv3619c/IFA alone significantly inhibited the OVA-induced up-regulation of pERK1/2 and was similar to PBS-challenged control mice. This inhibition of ERK1/2 may be related to inhibition of IL-5 and other Th2 cytokines secretion., since it has been reported to be a downstream signaling molecule for many of the Th2 cytokines (Lui et al., 2010). Furthermore, treatment with both low (0.5 mg) and high dose (3 mg) dexamethasone inhibited pERK1/2, consistent with data from previous studies (Liu et al., 2013; El-Hashim et al., 2017). This suggests that Rv3619c/IFA and glucocorticoids may at least share some of the mechanisms that result in the inhibition of molecular cell signaling pathways seen in allergic inflammation.

## Summary

The data presented in this study show that, in a murine asthma model, immunization with Rv3619c/IFA effectively inhibits the OVA-induced total cell counts, eosinophil airway cell infiltration in BAL fluid, perivascular and peribronchial inflammation and fibrosis and goblet cell hyper/metaplasia, and was as effective as treatment with a high dose of steroid. In addition, Rv3619c/IFA inhibited the OVA-induced elevated IL-5 levels in spleen cells, OVA-specific IgE, IgG and IgG1 levels in sera, and pERK1/2 expression in lung tissue. This study demonstrates that Rv3619c/IFA may be an effective vaccine for the prevention of asthma.

## Data Availability Statement

All datasets generated for this study are included in the article/**Supplementary Material**.

## Ethics Statement

The animal study was reviewed and approved by Health Science Center Animal Welfare Committee, Kuwait University.

## Author Contributions

Conceptualization: AM, HAS, HA, AE-H. Data curation: HAS, AM, AE-H. Formal analysis: AM, HAS, HA, AE-H. Funding acquisition: AM, HA, AE-H, HAS. Investigation: HAS, AM, HA, AE-H. Methodology: HAS, AM, AE-H. Project administration: AM. Resources: AM, AE-H. Software: HAS. Supervision: AM, HA, AE-H. Validation: HAS, AM, HA, AE-H. Visualization: HAS, AM, HA, AE-H. Writing–original draft: HAS. Writing–review and editing: AM, HA, AE-H.

## Funding

This work was supported by the College of Graduate Studies and the Research Sector Projects YM06/15 and SRU02/13 of Kuwait University, and partially funded by Kuwait Foundation for the Advancement of Sciences (KFAS) under project code CB17-63MM-03.

## Conflict of Interest

The authors declare that the research was conducted in the absence of any commercial or financial relationships that could be construed as a potential conflict of interest.

## Supplementary Material

Supplementary Figure 1 SDS-anaylsis of Rv3619c protein purified from GST fusion partner. Lane M: Low molecular weight marker, Lanes 1-3: free Rv3619c protein (9.8 kDa).

## References

[B1] Ahmad S. Ali M. M. Mustafa A. S. (2003). Construction of a modified vector for efficient purification of recombinant *Mycobacterium tuberculosis* proteins expressed in Escherichia coli. Protein Expr. Purif. 29, 167–175. 10.1016/S1046-5928(03)00052-4 12767806

[B2] Alam R. Gorska M. M. (2011). MAPK signaling and ERK1/2 bistability in asthma. Clin. Exp. Allergy 41, 149–159. 10.1111/j.1365-2222.2010.03658.x 21121982 PMC3115726

[B3] Baena-Cagnani C. E. Teijeiro A. Canonica G. W. (2015). Four-year follow-up in children with moderate/severe uncontrolled asthma after withdrawal of a 1-year omalizumab treatment. Curr. Opin. Allergy Clin. Immunol. 15, 267–271. 10.1097/ACI.0000000000000161 25899697

[B4] Chen J. B. Ramadani F. Pang M. O. Y. Beavil R. L. Holdom M. D. Mitropoulou A. N. (2018). Structural basis for selective inhibition of immunoglobulin E-receptor interactions by an anti-IgE antibody. Sci. Rep. 8, 11548. 10.1038/s41598-018-29664-4 30069035 PMC6070508

[B5] Cookson W. O. Moffatt M. F. (1997). Asthma: an epidemic in the absence of infection?. Science 275, 41–42. 10.1126/science.275.5296.41 8999535

[B6] Drick N. Seeliger B. Welte T. Fuge J. Suhling H. (2018). Anti-IL-5 therapy in patients with severe eosinophilic asthma – clinical efficacy and possible criteria for treatment response. BMC Pulm. Med. 18, 119. 10.1186/s12890-018-0689-2 30021546 PMC6052600

[B7] El-Hashim A. Z. Renno W. M. Raghupathy R. Abduo H. T. Akhtar S. Benter I. F. (2012). Angiotensin-(1-7) inhibits allergic inflammation, via the MAS1 receptor, through suppression of ERK1/2- and NF-κB-dependent pathways. Br. J. Pharmacol. 166, 1964–1976. 10.1111/j.1476-5381.2012.01905.x 22339213 PMC3402818

[B8] El-Hashim A. Z. Khajah M. A. Renno W. M. Babyson R. H. Uddin M. Benter I. F. (2017). Src-dependent EGFR transactivation regulates lung inflammation via downstream signaling involving ERK1/2, PI3Kδ/ Akt and NFκB induction in a murine asthma model. Sci. Rep. 7, 9919. 10.1038/s41598-017-09349-0 28855674 PMC5577320

[B9] Gharagozloo M. Jafari S. Esmaeil N. Javid E. N. Bagherpour B. Rezaei A. (2013). Immunosuppressive effect of Silymarin on mitogen-activated protein kinase signalling pathway: the impact on t cell proliferation and cytokine production. Basic Clin. Pharmacol. Toxicol. 113, 209–214. 10.1111/bcpt.12088 23701595

[B10] Goplen N. Gorska M. M. Stafford S. J. Rozatio S. Guo L. Liang Q. (2008). A phosphosite screen identifies autocrine TGF-beta-driven activation of protein kinase R as a survival-limiting factor for eosinophils. J. Immunol. 180, 2456–2464. 10.4049/jimmunol.180.6.4256 18322238

[B11] Guan X. J. Zhang W. X. Li C. C. Zheng Y. M. Lin L. Ye L. P. (2007). The role of external signal regulated kinase and transforming growth factor beta(1) in asthma airway remodeling and regulation of glucocorticoids. Zhonghua Yi Xue Za Zhi 87 (25), 1767–1772. 17919386

[B12] Han E. R. Choi I. S. Eom S. H. Kim H. J. (2010). Preventive effects of mycobacteria and their culture supernatants against asthma development in BALB/c mice. Allergy Asthma Immunol. Res. 22, 34–40. 10.4168/aair.2010.2.1.34 PMC2831609 20224676

[B13] Han E. R. Choi I. S. Choi H. G. Kim H. J. (2012). Therapeutic effects on mycobacterial secretory proteins against established asthma in BALB/c Mice. Allergy Asthma Immunol. Res. 4, 214–221. 10.4168/aair.2012.4.4.214 22754715 PMC3378928

[B14] Hanif S. N. Al-Attiyah R. Mustafa A. S. (2010a). Molecular cloning, expression, purification and immunological characterization of three low-molecular weight proteins encoded by genes in genomic regions of difference of. Mycobacterium Tubercul. Scand. J. Immunol. 71, 353–361. 10.1111/j.1365-3083.2010.02388.x 20500686

[B15] Hanif S. N. M. Al-Attiyah R. Mustafa A. S. (2010b). DNA vaccine constructs expressing *Mycobacterium tuberculosis*-specific genes induce immune responses. Scand. J. Immunol. 72, 408–415. 10.1111/j.1365-3083.2010.02452.x 21039735

[B16] Harati E. Bahrami M. Razavi A. Kamalinejad M. Mohammadian M. Rastegar T. (2018). Effects of viola tricolor flower hydroethanolic extract on lung inflammation in a mouse model of chronic asthma. Iran J. Allergy Asthma Immunol. 17 (5), 409–417. 10.18502/ijaai.v17i5.299 30518183

[B17] Hosoki K. Ying S. Corrigan C. Qi H. Kurosky A. Jennings K. (2015). Analysis of a panel of 48 cytokines in BAL fluids specifically identifies IL-8 levels as the only cytokine that distinguishes controlled asthma from uncontrolled asthma, and correlates inversely with FEV1. PLoS One 10, e0126035. 10.1371/journal.pone.0126035 26011707 PMC4444276

[B18] Ji N. F. Xie Y. C. Zhang M. S. Zhao X. Cheng H. Wang H. (2014). Ligustrazine corrects Th1/Th2 and Treg/Th17 imbalance in a mouse asthma model. Int. Immunopharmacol. 21, 76–81. 10.1016/j.intimp.2014.04.015 24785327

[B19] Karamatsu K. Matsuo K. Inada H. Tsujimura Y. Shiogama Y. Matsubara A. (2012). Single systemic administration of Ag85B of mycobacteria DNA inhibits allergic airway inflammation in a mouse model of asthma. J. Asthma Allergy 5, 71–79. 10.2147/JAA.S37667 23271916 PMC3526857

[B20] Khajah M. A. Fateel M. M. Ananthalakshmi K. V. Luqmani Y. A. (2016). Anti-inflammatory action of angiotensin 1-7 in experimental colitis. PLoS One 11(3), e0150861. 10.1371/journal.pone.0150861 26963721 PMC4786309

[B21] Koch M. Witzenrath M. Reuter C. Herma M. Schütte H. Suttorp N. (2006). Role of local pulmonary IFN-gamma expression in murine allergic airway inflammation. Am. J. Respir. Cell Mol. Biol. 35 (2), 211–219. 10.1165/rcmb.2005-0293OC 16543606

[B22] Krishna M. T. Salvi S. S. (2002). Could administration of bacille CalmetteGue´rin vaccination at birth protect from the development of asthma and allergic diseases in the western world? Has this question been adequately investigated? Pediatr. Allergy Immunol. 13 (3), 172–176. 10.1034/j.1399-3038.2002.01048.x 12144638

[B23] Krug N. Madden J. Redington A. E. Lackie P. Djukanovic R. Schauer U. (1995). T-cell cytokine profile evaluated at the single cell level in BAL and blood in allergic asthma. Am. J. Respir. Cell Mol. Biol. 14 (4), 319–326. 10.1165/ajrcmb.14.4.8600935 8600935

[B24] Kumar R. K. Yang M. Herbert C. Foster P. S. (2012). Interferon-γ, pulmonary macrophages and airway responsiveness in asthma. Inflammation Allergy Drug Targets 11 (4), 292–297. 10.2174/187152812800958951 22856520

[B25] Kurakula K. Vos M. Logiantara A. Roelofs J. Nieuwenhuis M. A. Koppelman G. H. (2015). Deficiency of FHL2 attenuates airway inflammation in mice and genetic variation associates with human bronchial hyper-responsiveness. Allergy 70, 1531–1544. 10.1111/all.12709 26222912

[B26] Kurvilla M. E. Vanijcharoenkarn K. Shih J. A. Lee F. E. H. (2019). Epidemiology and risk factors for asthma. Respir. Med. 149, 16–22. 10.1016/j.rmed.2019.01.014 30885424

[B27] Liu J. Zhang M. Niu C. Luo Z. Dai J. Wang L. (2013). Dexamethasone inhibits repair of human airway epithelial cells mediated by glucocorticoid-induced leucine zipper (GILZ). PLoS One 8, e60705. 10.1371/journal.pone.0060705 23573276 PMC3615997

[B28] Lui W. Tundwal K. Liang Q. Goplen N. Rozario S. Quayum N. (2010). Establishment of extracellular signal-regulated kinase 1/2 bistability and sustained activation through Sprouty 2 and its relevance for epithelial function. Mol. Cell Biol. 30, 1783–1799. 10.1128/MCB.01003-09 20123980 PMC2838067

[B29] Ma X. Ma Z. Wang J. Sun Z. Yu W. (2014). Effect of Hyssopus officinalis L. on inhibiting airway inflammation and immune regulation in a chronic asthmatic mouse model. Exp. Ther. Med. 8, 1371–1374. 10.3892/etm.2014.1978 25289025 PMC4186396

[B30] Mahmood A. Srivastava S. Tripathi S. Ansari M. A. Owais M. Arora A. (2011). Molecular characterization of secretory proteins Rv3619c and Rv3620c from *Mycobacterium tuberculosis* H37Rv. FEBS J. 278, 341–353. 10.1111/j.1742-4658.2010.07958.x 21134129

[B31] Mangtani P. Abubakar I. Ariti C. Beynon R. Pimpin L. Fine P. E. (2014). Protection by BCG vaccine against tuberculosis: a systematic review of randomized controlled trials. Clin. Infect. Dis. 58, 470–480. 10.1093/cid/cit790 24336911

[B32] Mansur A. H. Srivastava S. Mitchell V. Sullivan J. Kasujee I. (2017). Longterm clinical outcomes of omalizumab therapy in severe allergic asthma: Study of efficacy and safety. Respir. Med. 124, 36–43. 10.1016/j.rmed.2017.01.008 28284319

[B33] Marciano B. E. Huang C. Y. Joshi G. Rezaei N. Carvalho B. C. Allwood Z. (2014). BCG vaccination in patients with severe combined immunodeficiency: complications, risks, and vaccination policies. J. Allergy Clin. Immunol. 133, 1134–1141. 10.1016/j.jaci.2014.02.028 24679470 PMC4015464

[B34] Matucci A. Vultaggio A. Maggi E. Kasujee I. (2018). Is IgE or eosinophils the key player in allergic asthma pathogenesis? Are we asking the right question? Resp. Res. 19, 113. 10.1186/s12931-018-0813-0 PMC5992661 29879991

[B35] McBrien C. N. Menzies-Gow A. (2017). The biology of eosinophils and their role in asthma. Front. Med. (Lausanne) 4, 93. 10.3389/fmed.2017.00093 28713812 PMC5491677

[B36] Mustafa A. S. Al-Attiyah R. (2009). Identification of *Mycobacterium tuberculosis*-specific genomic regions encoding antigens including protective cellular immune responses. Indian J. Exp. Biol. 47, 498–504. 19634716

[B37] Obihara C. C. Kimpen J. L. Beyers N. (2007). The Potential of *Mycobacterium* to protect against allergy and asthma. Curr. Allergy Asthma Rep. 7, 223–230. 10.1007/s11882-007-0076-1 17448335

[B38] Olin J. T. Wechsler M. E. (2014). Asthma: pathogenesis and novel drugs for treatment. BMJ 24;349, g5517. 10.1136/bmj.g5517 25420994

[B39] Ownby D. R. Johnson C. C. (2009). “Factors underlying the increasing incidence and prevalence of allergic diseases,” in Middleton"s allergy: principles & practice, 7th ed. Eds. Adkinson N. F. Jr Bochner B. S. Busse W. W. Holgate S. T. Lemanske R. F. Jr Simons F. E. (Philadelphia: Mosby), 769–778. 2.

[B40] Pelaia C. Vetrella A. Busceti M. T. Gallelli L. Terracciano R. Savino R. (2017). Severe eosinophilic asthma: from the pathogenic role of interleukin-5 to the therapeutic action of mepolizumab. Drug Des. Devel. Ther. 11, 3137–3144. 10.2147/DDDT.S150656 PMC5669784 29133975

[B41] Pennington L. F. Tarchevskaya S. Brigger D. Sathiyamoorthy K. Graham M. T. Nadeau K. C. (2016). Structural basis of omalizumab therapy and omalizumab-mediated IgE exchange. Nat. Commun. 7, 11610. 10.1038/ncomms11610 27194387 PMC4873975

[B42] Raundhal M. Morse C. Khare A. Oriss T. B. Milosevic J. Tradeau J. (2015). High IFN-γ and low SLPI mark severe asthma in mice and humans. J. Clin. Invest. 125 (8), 3037. 10.1172/JCI80911 26121748 PMC4563754

[B43] Richards J. D. Dave S. H. Chou C. H. Mamchak A. A. DeFranco A. L. (2001). Inhibition of the MEK/ERK signaling pathway blocks a subset of B cell responses to antigen. J. Immunol. 166, 3855–3864. 10.4049/jimmunol.166.6.3855 11238629

[B44] Safar H. A. Mustafa A. S. Amoudy H. A. El-Hashim A. (2020). The effect of adjuvants and delivery systems on Th1, Th2, Th17 and Treg cytokine responses in mice immunized with *mycobacterium tuberculosis*-specific proteins. PLoS One 15 (2), e0228381. 10.1371/journal.pone.0228381 32027660 PMC7004338

[B45] Shaban K. Amoudy H. A. Mustafa A. S. (2013). Cellular immune responses to recombinant *Mycobacterium bovis* BCG constructs expressing major antigens of region of difference 1 of *Mycobacterium tuberculosis*. Clin. Vaccine Immunol. 20, 1230–1237. 10.1128/CVI.00090-12 23761657 PMC3754513

[B46] Sharma S. Rasal V. P. Patil P. A. Joshi R. K. (2017). Effect of Angelica glauca essential oil on allergic airway changes induced by histamine and ovalbumin in experimental animals. Indian J. Pharmacol. 49, 55–59. 10.4103/0253-7613.201019 28458423 PMC5351239

[B47] Shrimanker R. Pavord I. D. (2017). Interleukin-5 inhibitors for severe asthma: rationale and future outlook. BioDrugs 31, 93–103. 28364396 10.1007/s40259-017-0215-8

[B48] Stokes J. (2017). Anti-IgE treatment for disorders other than asthma. Front. Med. (Lausanne) 4, 152. 28983485 10.3389/fmed.2017.00152 PMC5613080

[B49] Tsujimura Y. Inada H. Yoneda M. Fujita T. Matsuo K. Yasutomi Y. (2014). Effects of mycobacteria major secretion protein, Ag85B, on allergic inflammation in the lung. PLoS One 9, e106807. 25192550 10.1371/journal.pone.0106807 PMC4156387

[B50] Wakashin H. Hirose K. Maezawa Y. Kagami S. Suto A. Watanabe N. (2008). IL-23 and Th17 cells enhance Th2-cell-mediated eosinophilic airway inflammation in mice. Am. J. Respir. Crit. Care Med. 178, 1023–1032. 10.1164/rccm.200801-086OC 18787221

[B51] Williams D. M. (2018). Clinical pharmacology of corticosteroids. Respir. Care 63, 655–670. 10.4187/respcare.06314 29794202

[B52] Wu J. Xu J. Cai C. Gao X. Li L. Zong N. (2009). Ag85B DNA vaccine suppresses airway inflammation in a murine model of asthma. Resp. Res. 10, 51. 10.1186/1465-9921-10-51 PMC2713210 19531238

[B53] Wuhao L. Ran C. Xujin H. Zhongdao W. Dekumyoy P. Zhiyue L. (2017). Parasites and asthma. Parasitol. Res. 116, 2373–2383. 10.1007/s00436-017-5548-1 28689246

[B54] Zhang J. Guo S. Li C. Jiang X. (2012). Therapeutic effects of inhaled inactivated *Mycobacterium phlei* in adult patients with moderate persistent asthma. Immunotherapy 4, 383–387. 10.2217/imt.12.25 22512632

[B55] Zhang T. Yang Z. Yang S. Du J. Wang S. (2015). Immunoregulatory effects of paeoniflorin exerts anti-asthmatic effects via modulation of the Th1/Th2 equilibrium. Inflammation 38, 2017–2025. 10.1007/s10753-015-0182-5 25971794

[B56] Zhang Y. Feng Y. Li L. Ye X. Wang J. Wang Q. (2018). Immunization with an adenovirus-vectored TB vaccine containing Ag85A-Mtb32 effectively alleviates allergic asthma. J. Mol. Med. (Berl.) 96, 249–263. 10.1007/s00109-017-1614-5 29302700 PMC5859035

[B57] Zhao J. Yeong L. H. Wong W. S. F. (2006). Dexamethasone alters bronchoalveolar lavage fluid proteome in a mouse asthma model. Int. Arch. Allergy Immunol. 142, 219–229. 10.1159/000097024 17108703

