# Supplementary figures and images for: Mycobacterium tuberculosis–Specific Antigen Rv3619c Effectively Alleviates Allergic Asthma in Mice
Source: Front Pharmacol. 2020 Sep 25;11:532199. doi: 10.3389/fphar.2020.532199 (PMC7546857; doi:10.3389/fphar.2020.532199)

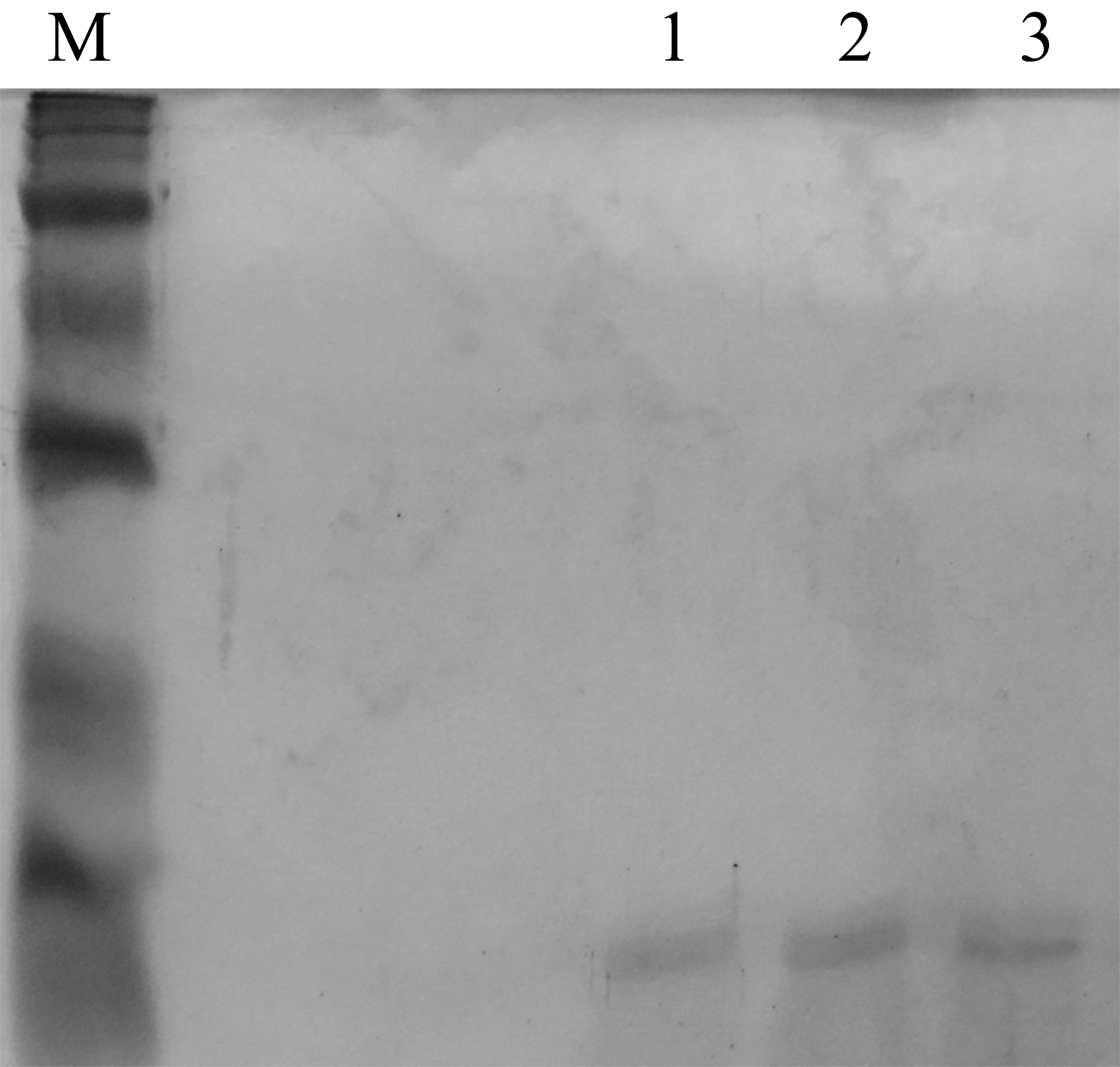

Supplement: Supplementary Figure 1 — SDS-anaylsis of Rv3619c protein purified from GST fusion partner. Lane M: Low molecular weight marker, Lanes 1-3: free Rv3619c protein (9.8 kDa). [file Image_1.tif]
